# STAT3 isoforms differentially affect ACE2 expression: A potential target for COVID‐19 therapy

**DOI:** 10.1111/jcmm.15838

**Published:** 2020-09-19

**Authors:** Inbal Shamir, Mor Abutbul‐Amitai, Haya Abbas‐Egbariya, Metsada Pasmanik‐Chor, Gideon Paret, Yael Nevo‐Caspi

**Affiliations:** ^1^ Department of Pediatric Critical Care Medicine Safra Children’s Hospital Sheba Medical Center Tel Hashomer Israel; ^2^ George S. Wise Faculty of Life Science Bioinformatics Unit Tel Aviv University Tel Aviv Israel; ^3^ Sackler Medical School Tel‐Aviv University Tel‐Aviv Israel

**Keywords:** ACE2, COVID‐19, SARS‐CoV‐2, STAT3 isoforms, STAT3α, STAT3β

## Abstract

The SARS‐coronavirus 2 is the aetiologic agent COVID‐19. ACE2 has been identified as a cell entry receptor for the virus. Therefore, trying to understand how the gene is controlled has become a major goal. We silenced the expression of STAT3α and STAT3β, and found that while silencing STAT3α causes an increase in ACE2 expression, silencing STAT3β causes the opposite effect. Studying the role of STAT3 in ACE2 expression will shed light on the molecular events that contribute to the progression of the disease and that the different roles of STAT3α and STAT3β in that context must be taken in consideration. Our results place STAT3 in line with additional potential therapeutic targets for treating COVID‐19 patients.

## 1 INTRODUCTION

SARS‐CoV‐2, detected in patients, is the aetiologic agent of COVID‐19.^1^ The virus causes symptoms ranging from mild disease to severe lung injury and multi‐organ failure, eventually leading to death. Considering the high mortality rate of COVID‐19, the development of effective therapeutics is an urgent issue and requires the identification of quality targets.

ACE2 (Angiotensin I Converting Enzyme 2), an important player in the renin‐angiotensin‐aldosterone system (RAAS),^2^, ^3^ has been identified as a cell entry receptor for SARS‐CoV‐2.^4^, ^5^ It is therefore not surprising that ACE2 has taken centre stage in the COVID‐19 outbreak and that numerous groups have been trying to understand how the gene is controlled and to search for methods to control its' expression.

We have been studying transcriptional regulation by STAT3 alternatively spliced isoforms: STAT3α, the main isoform, and STAT3β which encodes a shorter protein, lacking the C terminal transactivation domain of STAT3α.^6^, ^7^ In this study, we show that both STAT3α and STAT3β affect the expression of ACE2, however, in a distinctive manner.

## 2 MATERIALS AND METHODS

MCF‐7 cells, from a human breast carcinoma, were obtained from the ATCC and cultured in DMEM with 10% FBS, 1% penicillin:streptomycin and 1% glutamine, at 37°C in a humidified incubator with 5% CO_2_.

SiRNAs are specific for each of the STAT3 isoforms (Ambion, Pleasanton, CA, USA) (5' to 3'):

siSTAT3α‐sense: GCAAUACCAUUGACCUGCCtt;
siSTAT3α‐antisense: GGCAGGUCAAUGGUAUUGCtg;
siSTAT3β‐sense: GUGUGACACCAUUCAUUGAtt;
siSTAT3β‐antisense: UCAAUGAAUGGUGUCACACag;
siRNA negative control (siCON) (Ambion #AM4635).

### 2.1 Transfection of siRNA

MCF‐7 cells were transfected using Lipofectamin 3000^®^ (Invitrogen, Carlsbad, CA, USA). 2.5 × 10^5^ cells were seeded in a six‐well plate. After 24 hours, transfection was continued according to the manufacturer's protocol. Cells were then incubated at 37°C for 4 hours followed by addition of one ml medium (siSTAT3α was added to a concentration of 0.02 µmol/L; siSTAT3β to a concentration of 0.01 µmol/L; siCON was added to a similar concentration as the relevant siSTAT3). After 48 hours, the expressions of mRNA and proteins were analysed. All experiments were repeated at least three times.

### 2.2 RNA extraction, cDNA preparation and PCR analysis

Total RNA was extracted using TRI Reagent^®^ (Sigma/Merck, Darmstadt, Germany) according to the manufacturer's protocol. cDNA was prepared from 2 µg RNA with the High Capacity cDNA Reverse‐Transcription Kit (Applied Biosystems, Vilnius, Lithuania).

Relative quantitative (RQ)‐PCR was performed with the TaqMan^®^ or SYBR Green^®^ Fast Advanced Master Mixes (Applied Biosystems) with 0.5 µmol/L specific primers and probes (5'FAM, 3'BHQ) (except for STAT3β used at 2.5 µmol/L):

STAT3α‐F: TGACACCAACGACCTGCAG.
STAT3α‐R: CAGCACCTTCACCATTATTTCCA.
STAT3α‐probe: CCCCGCACTTTAGATTCATTGATGCAGT.
STAT3β‐F: GCCCCATACCTGAAGACCAA.
STAT3β‐R: TCAGCACCTTCACCATTATTTCC.
STAT3β‐probe: TTTATCTGTGTGACACCATTCATTGATGCAGTT.
ABL‐F: TTTATCTGTGTGACACCATTCATTGATGCAGTT.
ABL‐R: GATGTAGTTGCTTGGGACCCA.
ABL‐probe: GATGTAGTTGCTTGGGACCCA.
ACE2‐F: ATGAAGGCCCTCTGCACAAA.
ACE2‐R: TTCCAAGCCTCAGCATATTGAAC.
ACE‐F: GCCAGATCTGACGAATGTGA.
ACE‐R: TCGGGTAAAACTGGAGGATG.

All primers enable the amplification of mature mRNA only. We used the ABL gene as the reference gene. All reactions (10 µL) were performed in triplicates on the Applied Biosystems StepOne™ machine using the StepOne v2.3 software. RQ analyses were performed with the ΔΔCT method. Each experiment was performed at least three times. Results were normalized to those with transfection of siCON that were set to one.

### 2.3 Cell lysis and Western blot analysis

Proteins were extracted using RIPA buffer with protease inhibitor (Roche, Basel, Switzerland). Following separation on a 7.5% SDS‐PAGE, proteins were transferred to a nitrocellulose membrane followed by staining with a primary antibody overnight at 4°C, washed and incubated with the appropriate secondary antibody for 45 minutes at room temperature. Specific reactive bands were detected using horseradish peroxidase‐conjugated secondary antibodies by enhanced chemiluminescence (Cyanagen). Quantification of proteins was performed with the ImageJ software. The antibodies used were as follows: anti‐STAT3 1:1000 (CST), anti‐ACE2 1:1000 (Abcam, Cambridge, UK) and anti‐αTubulin 1:30000 (Abcam).

### 2.4 Statistical analysis

*T* test was used to calculate statistical differences between two samples. Results are given as mean value ± SD. In graphs, columns marked with asterisks are significantly different then the control sample (*P ≤ 0.05; **P ≤ 0.01; ***P ≤ 0.001).

## 3 RESULTS

**FIGURE 1:**
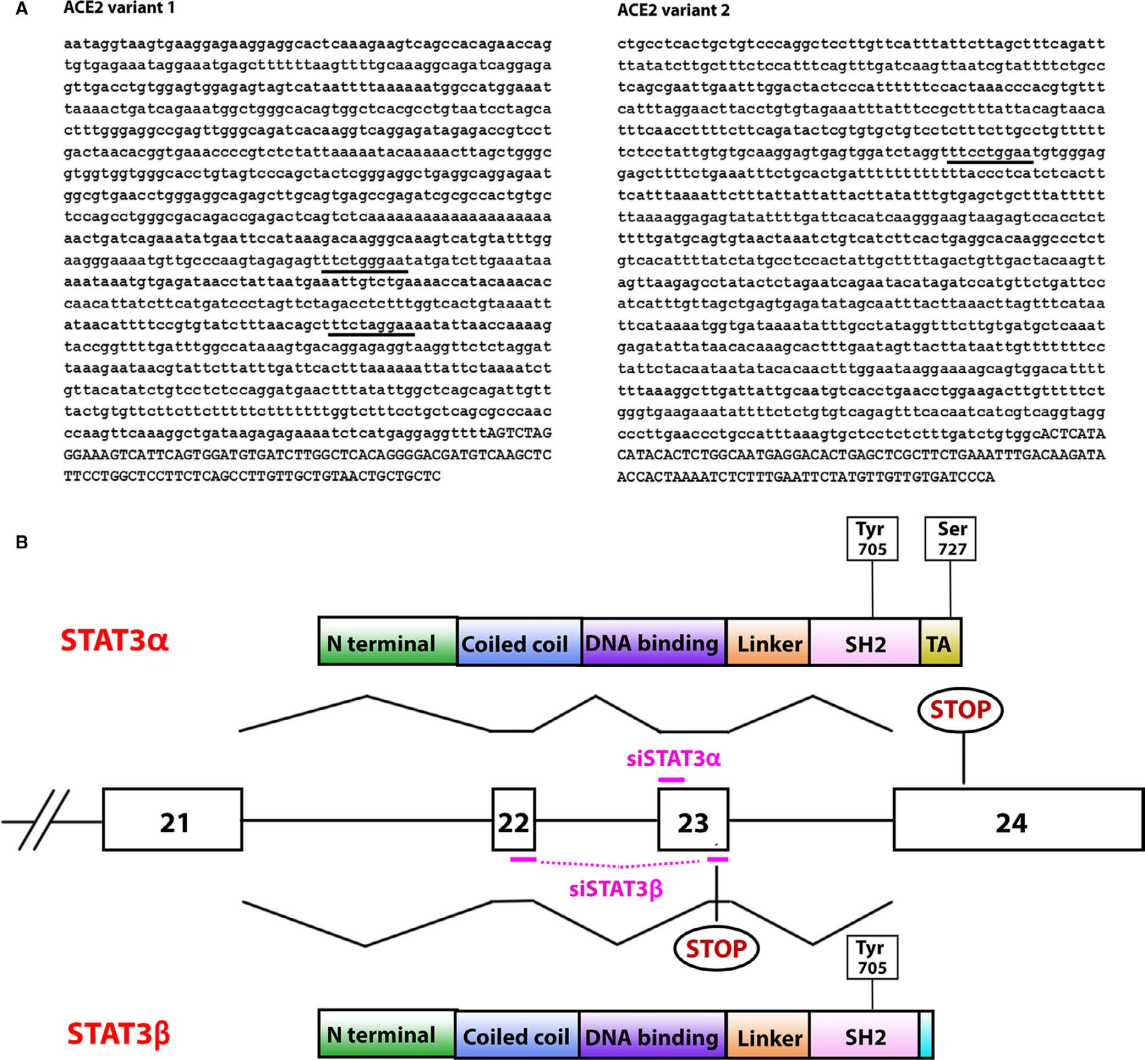
(A) Putative STAT3 binding sites in the ACE2 promoters ACE2 promoter sequences from −1000 bp to +100 bp relative to the transcription start site (TSS). Lower case letters are upstream TSS. Underlined bases mark the STAT3 putative binding sites. (B) STAT3 gene, transcripts and proteins organization Schematic representation of STAT3 exons 21‐24 and schematic overview of STAT3α and STAT3β protein domains and their derivation by normal or alternative splicing. Phosphorylation sites are marked. Stop codons of both proteins are marked. siRNA molecules are depicted in pink

ACE2 is encoded from two transcript variants. Examination of their promoters (EPD—Eukaryotic Promoter Database^8^) revealed that they both include canonical STAT3 binding motifs (TTCNNNGAA) (Figure 1A) suggesting that STAT3 is able to interact with the ACE2 promoter and to play a role in the regulation of ACE2 expression.

**FIGURE 2:**
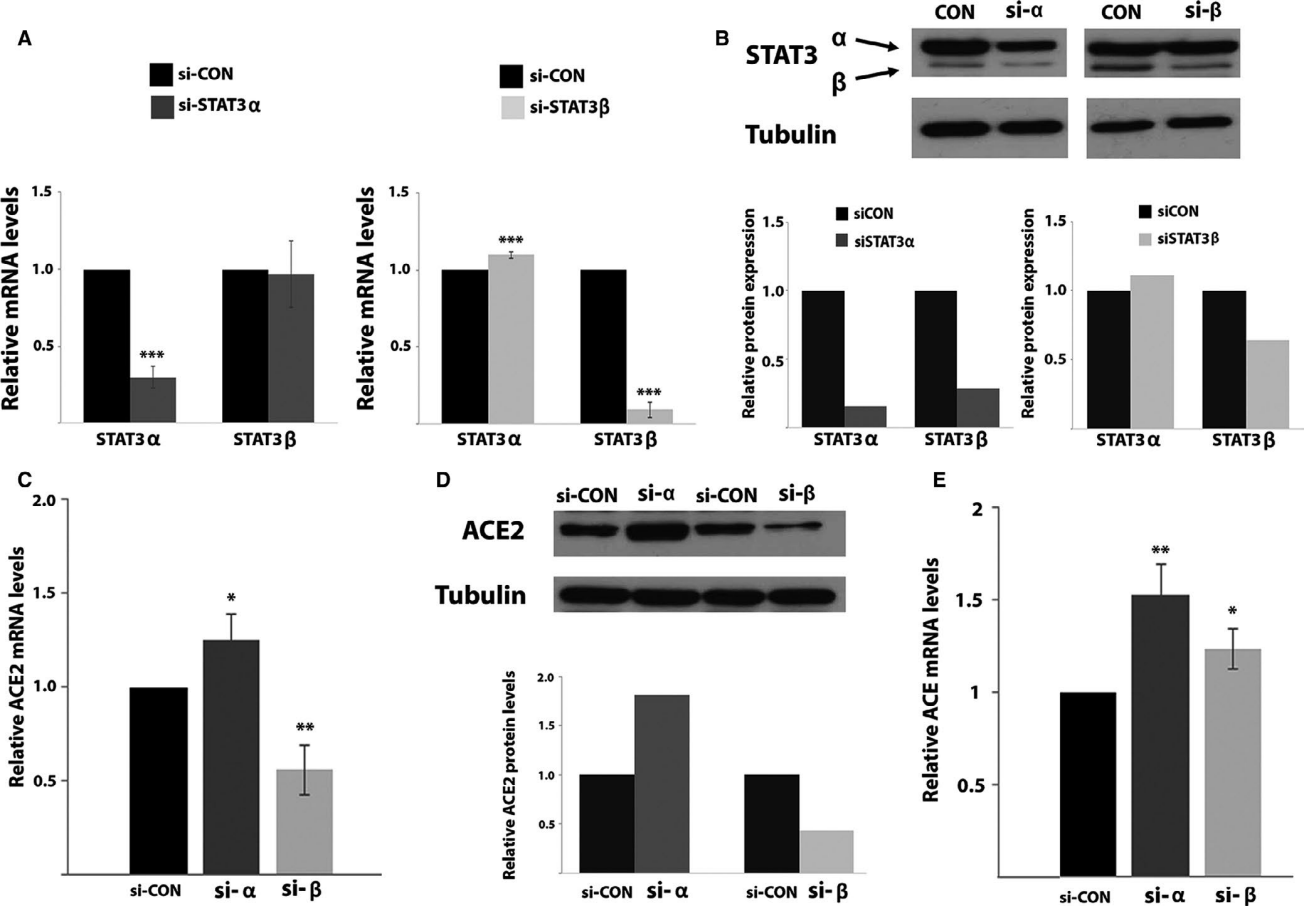
(A) Relative expression of the mRNA of STAT3 isoforms evaluated by RQ‐PCR. Results were normalized to those obtained upon transfection with the relevant negative control siRNA that were set to 1. Results are the mean of at least three repeats of each experiment ±SD. ***P ≤ 0.001. (B) Western blot analysis of cells transfected with the indicated siRNA. Proteins were probed with an anti‐STAT3 or anti‐αTubulin antibody. Relative protein quantification was performed with the ImageJ software. The protein amount obtained in the relevant control transfection was set to 1. (C) ACE2 mRNA relative expression levels evaluated by RQ‐PCR. Results were normalized to those obtained upon transfection with the relevant negative control siRNA that were set to 1. Results are the mean of at least three repeats of each experiment ±SD. *P ≤ 0.05; **P ≤ 0.01. (D) Western blot analysis of cells transfected with the indicated siRNA. Proteins were probed with an anti‐ACE2 or anti‐αTubulin antibody. Relative protein quantification was performed with the ImageJ software. The protein amount obtained in the relevant control transfection was set to 1. (E) ACE mRNA levels upon silencing of STAT3α or STAT3β ACE mRNA relative expression levels evaluated by RQ‐PCR. Results were normalized to those obtained upon transfection with the relevant negative control siRNA that were set to 1. Results are the mean of at least three repeats of each experiment ±SD. *P ≤ 0.05; **P ≤ 0.01

To study the involvement of STAT3 in regulating ACE2 expression, we silenced separately STAT3α and STAT3β (Figure 1B) in MCF‐7 cells using specific siRNAs. RQ‐PCR analysis revealed that the siRNAs were isoform‐specific (Figure 2A). Western blot analysis revealed that upon STAT3α silencing both STAT3α and STAT3β protein levels decreased although STAT3α levels were decreased to a larger extent. STAT3β silencing resulted in a decrease in STAT3β protein expression and did not affect STAT3α expression (Figure 2B). These results suggest that in MCF7 cells reducing STAT3α protein levels causes a reduction in STAT3β expression; however, low STAT3β expression does not affect the expression of STAT3α.

ACE2 mRNA and protein levels were studied in MCF7 cells following transfection with either siSTAT3α or siSTAT3β. SiSTAT3α caused an *increase* in ACE2 mRNA and protein expression; however, siSTAT3β caused a *decrease* in ACE2 mRNA and protein levels (Figure 2C‐D) implying that STAT3 plays a role in controlling ACE2 expression: STAT3α and STAT3β proteins do so differently, resulting in opposite results in the context of ACE2 expression.

This differential effect was not observed for ACE. Transfection with siSTAT3α or siSTAT3β in MCF7 cells resulted in an increase in ACE mRNA suggesting that both these proteins are involved in keeping low levels of ACE in the cells (Figure 2E).

## 4 DISCUSSION

Because of the outbreak of COVID‐19, caused by SARS‐CoV‐2 virus, there has been a race to elucidate the mechanisms for disease progression and identify treatment options for this novel pandemic disease. Extensive research is being devoted to identify proteins as targets for therapeutic agents. ACE2 is one such protein that has drawn considerable attention. Modifying the expression of ACE2 is a promising avenue for COVID‐19 therapy. We show that STAT3α, the main STAT3 protein, and STAT3β, a truncated STAT3 protein, affect the expression of ACE2 differently. To the best of our knowledge, this is the first study suggesting that STAT3 isoforms play a role in controlling ACE2 expression. Our study adds an additional member to the list of genes shown to be differentially regulated by the STAT3 isoforms.^9^ In most cells, the relative STAT3α:STAT3β protein levels are 4:1^10^ and therefore many studies ignore STAT3β and refer to STAT3α as a single STAT3 protein. We add an additional piece, to the already growing evidence regarding the unique role of STAT3β. Indeed, STAT3β has already been shown to act as an active transcriptional modulator.^11^, ^12^ In the setting of cancer, STAT3β has been shown to have a distinct, sometimes opposing, effect to STAT3α.^6^, ^13^

Several groups have suggested manipulating ACE2 levels as a therapeutic approach for COVID19.^14^, ^15^ While the effectiveness of such manipulations should be tested, elucidating the factors that affect ACE2 expression remains a major goal. Studying the role of STAT3 in ACE2 expression, with reference to the subtleties of the different roles of STAT3α and STAT3β in that context, will shed light on the molecular events that contribute to the progression of the disease and will enable examination of the possibility that these isoforms may serve as therapeutic targets for controlling SARS‐CoV‐2 entry.

## CONFLICT OF INTEREST

The authors confirm that there are no conflicts of interest.

## AUTHOR CONTRIBUTION

**Inbal Shamir:** Conceptualization (supporting); Data curation (equal); Investigation (equal); Writing‐review & editing (supporting). **Mor Abutbul‐Amitai:** Investigation (equal). **Haya Abbas‐Egbariya:** Investigation (supporting). **Metsada Pasmanik‐Chor:** Data curation (equal); Formal analysis (equal). **Gideon Paret:** Resources (lead); Supervision (supporting). **Yael Nevo‐Caspi:** Conceptualization (equal); Investigation (lead); Methodology (lead); Project administration (lead); Supervision (lead); Validation (supporting); Writing‐original draft (lead).
